# Awake oral behaviors associated with temporomandibular-related pain symptoms in a non-patient student population—a dual assessment approach

**DOI:** 10.22514/jofph.2025.048

**Published:** 2025-09-12

**Authors:** Alona Emodi-Perlman, Anna-Yael Czygrinow, Daniele Manfredini, Alessandro Bracci, Noa Ventura, Ilana Eli

**Affiliations:** ^1^Departement of Oral Rehabilitation, The Maurice and Gabriela Golddschleger School of Dental Medicine, Gray Faculty of Medicine and Health Sciences, Tel Aviv University, 69978 Tel Aviv, Israel; ^2^Orofacial Pain Unit, Department of Medical Biotechnologies, School of Dentistry, University of Siena, 53100 Siena, Italy; ^3^Department of Neurosciences, University of Padova, 35122 Padova, Italy

**Keywords:** Awake oral behaviors, Oral behavior checklist, Ecological momentary assessment, Masticatory muscle pain

## Abstract

**Background**: It is unclear which oral behaviors are harmless and which might become harmful when performed excessively. This study aimed to determine which awake oral behaviors are associated with Temporomandibular Disorder (TMD)-related pain symptoms in a non-patient population. **Methods**: Subjects’ awake oral behaviors were assessed through: (i) Oral Behavior Checklist (OBC), a single-point self-report questionnaire that quantifies the frequency of awake functional and non-functional oral behaviors; and (ii) Ecological Momentary Assessment (EMA), a designated smartphone application which enabled real-time repeated data collection of oral behaviors throughout the day, for multiple days. 118 participants (62.7% female) completed both the OBC and EMA assessment modes. Subjects were allocated into two groups: (i) subjects with TMD-related pain symptoms (TMDPS, N = 34, 85.29% female); and (ii) subjects without TMD-related pain symptoms (noTMDPS, N = 84, 53.57% female). **Results**: TMDPS subjects performed more awake oral behaviors compared to noTMDPS subjects. EMA behaviors that showed a significant predictive ability of masticatory muscle pain in binomial logistic regressions were entered into a multiple logistic regression model. Results show that teeth grinding increased the odds of subjects belonging to the TMDPS group by 22%. The final logistic regression demonstrated acceptable fit (Nagelkerke’s *R*^2^ = 0.232). In a multiple regression model evaluating the predictive ability of OBC behaviors, the final model showed a Nagelkerke’s *R*^2^ of 0.468. In addition to sex, three OBC behaviors came out as significant predictors in the final model: teeth grinding increased the odds of subjects belonging to the TMDPS group by 85%; holding or jutting the jaw increased the odds by 82%, and clenching increased the odds by 67%. **Conclusions**: Reports of teeth clenching, holding or jutting jaw forward or to the side, and teeth grinding may be associated with the report of TMD-related pain symptoms in a non-patient student population.

## 1. Introduction

Oral behaviors refer to a variety of activities involving the masticatory system [1, 2]. Yap *et al*. [3] classified awake oral behaviors into functional and non-functional types using the Oral Behavior Checklist (OBC). Functional awake oral behaviors are natural functions essential for daily life, including mastication, speaking, swallowing and breathing. In contrast, non-functional awake oral behaviors include awake bruxism behaviors (*e.g.*, teeth grinding, teeth clenching, teeth contact, bracing of the mandible or trusting of the mandible) and activities such as excursive positioning, object biting, and tongue pushing [4].

An international consensus on the assessment of bruxism summarized that: (i) sleep bruxism occurs during sleep and can be rhythmic or non-rhythmic, while awake bruxism occurs during wakefulness and is characterized by repetitive or sustained tooth contact, bracing or thrusting of the mandible; (ii) bruxism should be considered a behavior rather than a disorder; (iii) it can be assessed through self-reports, ecological momentary assessment (EMA), and instrumental approaches, such as electromyography (EMG); and (iv) bruxism-related masticatory muscle activities should be evaluated along a behavioral continuum [5, 6]. A recent systematic review [7] reported that the global prevalence of bruxism (both sleep and awake) is approximately 22%, with sleep bruxism and awake bruxism prevalence being 21% and 23%, respectively. An ongoing debate persists regarding whether normal functional and non-functional behaviors may become harmful when performed excessively [4, 8, 9, 10].

Other orofacial conditions with a relatively high prevalence include temporomandibular disorders (TMD). TMD is an umbrella term encompassing conditions that affect the temporomandibular joints (TMJ), masticatory muscles and related structures. These conditions are characterized by musculoskeletal pain and functional limitations, and/or acoustic sounds arising from the jaw joint [11]. The overall prevalence of TMD is approximately 31% among adults and elderly individuals, and 11% among children and adolescents, with regional variations observed: South America (47%), Asia (33%), and Europe (29%) [12, 13]. TMD is commonly assessed through a dual-axis approach, which considers both physiological factors (Axis I) and psychosocial dimensions (Axis II). It is well documented that psychosocial factors play a significant role in the development and progression of both TMD and bruxism [14]. Non-functional waking-state oral behaviors, including awake bruxism, have been found to correlate with psychological distress, with studies demonstrating a dose-response relationship—higher distress levels being associated with increased reports of oral behaviors [15].

Results from a recent meta-analysis [16] indicated a significant association between bruxism and TMD, with the presence of bruxism increasing the odds of developing TMD by 2.06–2.51 times (based on the type of bruxism). However, it is noteworthy that findings in the literature regarding the bruxism—TMD relationship vary significantly based on the assessment methods employed by researchers [17].

Even though the specific mechanisms linking different types of oral behaviors to TMD remain under investigation, these behaviors have been identified as a risk factor for the development of painful TMD [4, 9, 18, 19]. The variability in findings within this field suggests that different oral behaviors may affect the masticatory muscles and the TMJ in different ways, indicating the need for further research to understand their clinical relevance.

An accepted and validated method to assess and study oral behaviors is through single-point self-report observation [5, 15]. A widely used questionnaire is the Oral Behavior Checklist (OBC), which is a part of the Diagnostic Criteria of Temporomandibular Disorders (DC/TMD AXIS II). The OBC quantifies the self-reported frequency of various oral behaviors performed during the preceding month [1].

An additional, recently introduced strategy is an Ecological Momentary Assessment (EMA), conducted via a designated smartphone application (BruxApp®; version 1.0.71, WMA Srl., Florence, Italy). EMA has been recommended by the Standardized Tool for the Assessment of Bruxism (STAB) as a valid method for the real-time report of awake oral behaviors [20]. The application offers significant advantages over a single-point self-report by enabling real-time, repeated data collection throughout subjects’ waking period, possibly for multiple days [21]. While single-point self-reports provide only a retrospective snapshot, EMA allows the recording of current behaviors or experiences at multiple time points along a continuum [21]. Users can immediately indicate their jaw condition, *e.g.*, relaxed muscles, teeth clenching, and tongue positioning, allowing for continuous, behavior-specific monitoring. Previous studies have shown significant positive associations between behaviors reported on OBC and those recorded through EMA, supporting the concurrent validity of both tools [21, 22, 23, 24].

The aim of the present study was (i) to assess the type and frequency of awake oral behaviors in a non-clinical adult population with and without TMD-related pain symptoms, and (ii) to evaluate which of the awake oral behaviors are associated with an increased likelihood of TMD-related pain symptoms among this population.

## 2. Materials and methods

### 2.1 Study population

The study population consisted of a convenience sample of students from Tel Aviv University, the largest academic institution in Israel. The university’s student body includes individuals from diverse ethnic, cultural and social backgrounds, including Jewish, Muslim, Christian, secular, and orthodox groups. To reduce the risk of selection bias, students were recruited from two distinct professional faculties: the Faculty of Medicine (School of Dental Medicine) and the Faculty of Engineering. Students were approached between January and May 2024. To minimize researcher bias, data collection was conducted independently by two students (AY-C and NV). Furthermore, to reduce knowledge bias, participants studying for a Doctor of Dental Medicine (DMD) degree were recruited during the pre-clinical phase of their studies, before being exposed to the topic of TMD. To ensure standardized participants’ information, all participants received the same demonstration and explanation regarding EMA technology and the nature of oral behaviors, as outlined in the following section. The EMA software was programmed to send 20 alerts per day at random intervals, to reduce expectation bias, *i.e.*, the risk that participants might alter their behavior in anticipation of a scheduled alert [25].

A total of 210 students agreed to participate in the study. Exclusion criteria included a history of TMD management, any documented neuromuscular or psychiatric diseases, and/or recent facial trauma.

### 2.2 Instruments

Participants were requested to complete self-report questionnaires and use an EMA application (Bruxapp®, WMA Srl., Florence, Italy) for at least seven consecutive days, as detailed in section 2.2.3.

#### 2.2.1 Self-reported questionnaires

Personal data:

Personal data included sociodemographic data (age, gender, education, *etc.*) and medical history.

Single-point assessment of concurrent awake oral behaviors:

The official Hebrew version of the Oral Behavior Checklist (OBC) from the Diagnostic Criteria for Temporomandibular Disorders (DC/TMD) Axis II was used [26]. The OBC is a 21-item self-report questionnaire that assesses the frequency of oral behaviors over the preceding month [1], with responses ranging from 0 (none of the time) to 4 (all of the time). Mean scores for each group were calculated by multiplying each response value by the number of respondents selecting it, summing these products, and dividing by the total number of respondents for that item, as recommended for Likert-scale summary statistics [27]. While Likert data are ordinal, this approach is commonly used when examining group-level trends in psychological and behavioral research.

Following Donnarumma *et al*. [4], the first two questions related to sleep-related oral behaviors were excluded from the analysis. The remaining 19 questions, used in this study, can be accessed at: https://inform-iadr.com/wp-content/uploads/2024/03/Oral-Behaviors-Checklist_English-7-19-08-checkboxes.pdf. The single-point assessment was carried out on the first day, prior to the EMA assessment (see below).

TMD pain screener:

The TMD pain screener, part of the DC/TMD Axis I self-report tools [11], was used in its official Hebrew version [28]. The instrument comprises questions that refer to pain in the jaw or temple area in the last 30 days, at rest, and/or during activities. The total score ranges from 0 to 7, with a score of ≥3 indicating the presence of potential TMD [29]. The 6-item TMD pain screener was found to have excellent content validity and be effective in detecting painful TMD in various situations [28].

Based on the TMD pain screener, participants were categorized into two groups:

TMD-related pain symptoms group (TMDPS): participants with a score ≥3.

No TMD-related pain symptoms group (noTMDPS): participants with a score <3.

#### 2.2.2 Ecological momentary assessment

The BruxApp® (WMA Srl., Florence, Italy) is an EMA application, designated for awake oral behavior frequency assessment. The use of BruxApp (BA) was based on the protocol described by Bracci *et al*. [25], and the official Hebrew version of the app was utilized in this study.

Following the recommendations of Nykänen *et al*. [30], all participants watched a tutorial video explaining how to use the application and recognize the different awake oral behaviors before starting the report. After completing the tutorial and signing the consent form, each participant received a unique ID code, along with an explanation of how to register on the application.

After registration, participants received 20 randomly timed alert sounds per day, during the period 7:00 to 22:00, for seven consecutive days and/or until completing 7 valid days of report. Alerts were randomly generated to avoid expectancy bias [25]. Participants were instructed not to report while eating or speaking. Upon receiving each alert, participants had 5 minutes to report their current jaw condition by selecting one of the following behavior categories:

Relaxed (BA-relaxed)—a condition of perceived relaxation of jaw muscles, where there is no tension in the jaws or face.

Mandible Bracing (BA-bracing)—a condition of clenching of the jaw without contact between the teeth. Muscles are tense, and the jaw is in a fixed and rigid position.

Teeth contact (BA-teeth contact)—teeth are in contact in a fixed position with light touching.

Teeth Clenching (BA-clenching)—clenching of the teeth with strong contact in a fixed position.

Teeth Grinding (BA-grinding)—teeth rub against each other with sliding movements. This movement can occur in any direction and with different intensities of muscle strength.

BA-tongue pushing: tongue forcibly pressing against the teeth.

BA-tongue placing: tongue positioned between the teeth.

BA-biting or chewing: biting/chewing on tongue, lips or cheeks.

BA-holding objects: holding or biting objects between the teeth.

BA-chewing gum: gum chewing.

As suggested by Colonna *et al*. [21], a valid day for inclusion in the analysis was defined as one with a minimum of 12 responded alerts.

The study was approved by the Ethical Committee of Tel-Aviv University (Approval No: 0006074-2), and written informed consent was obtained from all participants.

### 2.3 Statistical analysis

Statistical analyses were performed with IBM SPSS Statistics (version 29.0, 2022, Armonk, NY, USA). Concisely, the frequencies (percentage) of each BruxApp condition were calculated as described by Bracci *et al*. [25]. The frequency of each condition or behavior was calculated as a percentage with respect to the answered alerts for all individuals. The frequencies were calculated daily on an individual basis, and individual frequencies were used to calculate an average for the study population daily. Data was reported as a range of mean values of the seven days, per condition.

As the OBC presents ordinal variables, nonparametric tests were used for comparisons between groups (Mann-Whitney U). Logistic regressions were conducted to assess the likelihood of participants having TMD-related pain symptoms. The significance level was set at *p* < 0.05. The internal consistency of self-report questionnaires was evaluated using Cronbach’s alpha, derived from an inter-item correlation matrix (reliability statistics).

## 3. Results

### 3.1 Descriptive results

Of the initial 210 students who consented to participate in the study, 150 completed the self-reported questionnaires and downloaded the EMA application. A total of 32 participants were excluded due to technical issues, failing to complete a minimum of 12 EMA alerts per day, or being unwilling to complete the study. Thus, the final study population comprised 118 participants (62.7% female). Among them, sixty-five students were enrolled in a DMD program (pre-clinical years), 26 students were pursuing a Bachelor of Science (BSc) degree in engineering, and 29 graduate students were enrolled in a Master of Science (MSc) degree in either of these disciplines.

The Cronbach’s α of the TMD pain screener, in the present study, was 0.846, indicating excellent internal consistency. A total of 34 of the participants were classified into the TMD-related pain symptoms group (TMDPS), with a mean age of 28.03 ± 3.27 years, of whom 85.29% were female. The remaining 84 participants belonged to the no TMD-related pain symptoms group (noTMDPS), with a mean age of 28.81 ± 3.68 years, and 53.57% were female.

A statistically significant difference was observed between the two groups in terms of sex distribution (*p* < 0.01, Chi-square test). No significant differences were found between groups for other sociodemographic variables.

### 3.2 Oral behavior checklist (OBC) results

The Cronbach’s α for the OBC items related to awake oral behaviors was 0.763, indicating acceptable internal consistency. The mean values of the OBC items ranged from 0.00 (OBC-play music) to 2.26 (OBC-eating).

Participants in the TMDPS group reported significantly higher frequencies of the following oral behaviors compared to the noTMDPS group: OBC-grinding, OBC-bracing, OBC-teeth contact, OBC-clenching, OBC-holding jaw, OBC-pressing tongue, OBC-bite or chew, OBC-hold or jut jaw, OBC-play music, OBC-lean with hand, and OBC-yawing. Moreover, there were strong effect sizes for the following behaviors: OBC-grinding, OBC-Bracing, OBC-Clenching, OBC-hold or jut jaw, and OBC-rigid jaw (Table 1).

**Table 1.** Comparison of OBC behaviors between TMDPS and noTMDPS groups (Mann-Whitney U).

Behavior	Group	Mean ± SD*	*p*	r**
OBC3: OBC-grinding				
	noTMDPS	**0.36 ± 0.67**	<0.001	0.39
	TMDPS	**1.26 ± 1.21**		
OBC6: OBC-bracing				
	noTMDPS	**1.14 ± 0.98**	<0.001	0.41
	TMDPS	**2.12 ± 0.98**		
OBC5: OBC-teeth contact				
	noTMDPS	**1.42 ± 1.08**	<0.001	0.27
	TMDPS	**2.09 ± 1.08**		
OBC4: OBC-clenching				
	noTMDPS	**0.96 ± 1.00**	<0.001	0.42
	TMDPS	**2.15 ± 1.23**		
OBC:7 OBC-hold or jut jaw				
	noTMDPS	**0.65 ± 0.83**	<0.001	0.40
	TMDPS	**1.65 ± 1.18**		
OBC8: OBC-press tongue				
	noTMDPS	**0.54 ± 0.88**	<0.001	0.25
	TMDPS	**1.06 ± 1.10**		
OBC9: OBC-tongue between teeth				
	noTMDPS	0.63 ± 0.89	NS	-
	TMDPS	1.06 ± 1.18		
OBC10: OBC-Bite or chew				
	noTMDPS	**1.10 ± 1.16**	0.043	0.19
	TMDPS	**1.47 ± 0.99**		
OBC11: OBC-rigid jaw				
	noTMDPS	**1.06 ± 1.11**	<0.001	0.39
	TMDPS	**2.06 ± 1.01**		
OBC12: OBC-hold objects				
	noTMDPS	0.63 ± 0.99	NS	-
	TMDPS	0.76 ± 1.16		
OBC13: OBC-chewing gum				
	noTMDPS	1.31 ± 1.12	NS	-
	TMDPS	1.24 ± 1.21		
OBC14: OBC-play music				
	noTMDPS	**0.00 ± 0.00**	0.006	0.25
	TMDPS	**0.18 ± 0.72**		
OBC15: OBC-lean with hand				
	noTMDPS	**1.58 ± 1.08**	0.031	0.2
	TMDPS	**2.06 ± 1.23**		
OBC16: OBC-chew one-sided				
	noTMDPS	0.85 ± 1.07	NS	-
	TMDPS	0.82 ± 1.03		
OBC17: OBC-eating				
	noTMDPS	1.99 ± 1.05	NS	-
	TMDPS	2.26 ± 1.16		
OBC18: OBC-talking				
	noTMDPS	0.83 ± 0.99	NS	-
	TMDPS	0.82 ± 1.09		
OBC19: OBC-singing				
	noTMDPS	0.49 ± 1.77	NS	-
	TMDPS	0.68 ± 0.98		
OBC20: OBC-yawning				
	noTMDPS	**1.64 ± 1.07**	0.006	0.25
	TMDPS	**2.09 ± 0.83**		
OBC21: OBC-telephone				
	noTMDPS	0.52 ± 0.74	NS	-
	TMDPS	0.65 ± 1.07		

*Significant differences marked in bold. **r—effect size. SD: standard deviation; OBC: Oral Behavior Checklist; TMDPS: TMD related pain symptoms; NS: Non-significant.

### 3.3 EMA results (BruxApp frequencies)

#### 3.3.1 Overall reporting frequencies

The total number of EMA reports among the entire study population (N = 118) was 10,927. The mean frequencies (based on a minimum of seven valid days of report per participant) ranged from 0.67 ± 2.84 for BA-holding objects to 51.23 ± 28.14 for BA-relaxed.

#### 3.3.2 Group comparisons: TMDPS *vs.* noTMDPS

Significant differences were observed between the TMDPS and noTMDPS groups in the frequency of certain EMA-reported behaviors (Fig. 1):

**Fig. 1.** Frequencies of EMA behaviors, according to the noTMDPS and TMDPS groups. Gray columns—noTMDPS, green columns—TMDPS; significant differences between groups marked in red bold, *p* < 0.05. TMDPS: TMD related pain symptoms; noTMDPS: no TMD-related pain symptoms.

(i) The noTMDPS group showed a significantly higher frequency of BA-relaxed behavior compared to the TMDPS group (55.24 ± 28.22 *vs.* 41.31 ± 25.93, respectively, *p* < 0.05, *r* = 0.23).

(ii) The TMDPS group showed a significantly higher frequency of BA-grinding (2.69 ± 5.25 *vs.* 0.61 ± 1.91, *p* < 0.01, *r* = 0.25) and of BA-tongue pushing (1.81 ± 3.07 *vs.* 0.76 ± 1.83; *p* < 0.01, *r* = 0.24) than the noTMDPS group. The difference between groups in BA-clenching was borderline (8.60 ± 10.49 *vs.* 5.87 ± 11.50; *p* = 0.065, *r* = 0.17).

#### 3.3.3 Changes in EMA-recorded behaviors over time

To evaluate the changes in EMA recorded behaviors over the seven-day observation period, a comparison between the initial day (Day 1) and the last day (Day 7) of data collection was performed using the Wilcoxon Signed Ranks Test.

In the TMDPS group, there was a significant increase in the frequency of the BA-relaxed behavior from Day 1 to Day 7 (32.83 ± 24.30 *vs.* 47.06 ± 37.09, *p* < 0.01, *r* = 0.45). In contrast, no such difference was observed in the noTMDPS group over the same period (Fig. 2).

**Fig. 2.** Mean daily frequencies of BA-relaxed behavior, in the noTMDPS and TMDPS groups. Orange line—noTMDPS, Green line—TMDPS. TMDPS: TMD related pain symptoms; noTMDPS: no TMD-related pain symptoms; BA: BruxApp.

No significant differences were found for any of the other EMA-assessed oral behaviors across the seven days.

### 3.4 Evaluation of the likelihood of subjects reporting TMD-related pain symptoms

To determine which EMA- and/or OBC behaviors could predict the presence of TMD-related pain symptoms, a series of logistic regression analyses were conducted as follows:

#### 3.4.1 Logistic regression analysis with EMA behaviors as independent variables

Each of the ten EMA behaviors was evaluated through a series of binomial logistic regression models. BA-relaxed, BA-grinding, and BA-tongue pushing showed significant predictive ability.

The final logistic regression model was conducted in two steps: first, age and sex were included as control variables (Nagelkerke *R*^2^ = 0.135); second, BA-tongue pushing and BA-grinding were added to the model. The final logistic regression model demonstrated acceptable fit (Nagelkerke *R*^2^ = 0.232). The model revealed that being female increased the odds of belonging to the TMDPC group 5.9 times. Whereas, BA-grinding increased the odds of subjects belonging to the TMDPC group by 22% (Table 2).

**Table 2.** Multiple logistic regression with EMA behaviors as independent variables—model coefficients.

Predictor	Estimate	S.E.	*Z*	*p*	Odds ratio	Lower	Upper
Intercept	−2.479	2.0075	−1.235	0.217	0.084	0.002	4.290
Gender: Female	1.787	0.588	3.0383	**0.002**	**5.969**	1.885	18.900
Age	5.97 × 10^−4^	0.0656	0.0091	0.993	1.001	0.880	1.140
BA-grinding	0.199	0.0745	2.6633	**0.008**	**1.220**	1.054	1.410

*Estimates represent the log odds of TMDPS vs. noTMDPS. Significant differences marked in bold. S.E.: Standard Error; BA: BruxApp.*

#### 3.4.2 Logistic regression analysis with OBC awake behaviors as independent variables

Each of the OBC awake behaviors was initially evaluated through simple logistic regression. Concisely, OBC-grinding, OBC-clenching, OBC-teeth contact, OBC-bracing, OBC-hold jaw, OBC-press tongue, OBC-tongue between teeth, OBC-rigid jaw, OBC-lean hand, and OBC-yawning demonstrated significant predictive ability and were therefore included in the multiple logistic regression model.

The final logistic regression model was also conducted in two steps: first, age and sex were entered as control variables. In the second step, OBC behaviors that showed a significant predictive ability were included in the model. Three OBC behaviors came out as significant predictors in the final model: OBC3-grinding, OBC4-clenching, and OBC7-hold or jut jaw. The model revealed that being a female increased the odds of belonging to the TMDPC group 5.7 times, teeth grinding increased the odds of belonging to the TMDPC group by 85%, holding or jutting jaw increased the odds by 82%, and clenching increased by 67% (Table 3). The final model demonstrated acceptable fit (Nagelkerke *R*^2^ = 0.468, Cox & Snell *R*^2^ = 0.327, McFadden *R*^2^ = 0.330). The values reflect relative improvement over a null model.

**Table 3.** Multiple logistic regression with OBC behaviors as independent variables—model coefficients.

Predictor	Estimate	S.E.	Wald	*df*	*p*	Odds ratio	95% Confidence Interval	
							Lower	Upper
Gender: Female	1.752	0.664	6.971	1	**0.008**	**5.765**	1.570	21.163
Age	−0.010	0.076	0.019	1	0.890	0.990	0.853	1.148
OBC3-Grinding	0.617	0.288	4.594	1	**0.032**	**1.853**	1.054	3.257
OBC4-Clenching	0.517	0.238	4.714	1	**0.030**	**1.676**	1.052	2.672
OBC7-Hold or jut jaw	0.599	0.275	4.733	1	**0.030**	**1.820**	1.061	3.122
Constant	−3.758	2.308	2.652	1	0.103	0.023		

*Estimates represent the log odds of TMDPS vs. noTMDPS. Significant results marked in bold. OBC: Oral Behavior Checklist; S.E.: Standard Error; df: degrees of freedom.*

Independent samples *t*-tests and Mann-Whitney U tests revealed significant differences between the TMDPS and non-TMDPS groups on all three OBC items included in the regression model: For grinding (OBC3), Welch’s *t* (41.4) = −4.11, *p* < 0.001, Cohen’s *d* = −0.93; For clenching (OBC4), Welch’s *t* (51.4) = −4.97, *p* < 0.001, Cohen’s *d* = −1.05; For holding or jutting jaw (OBC7), Welch’s *t* (47.3) = −4.47, *p* < 0.001, Cohen’s *d* = −0.97. Mann-Whitney U tests corroborated these findings (all *p*-values < 0.001), with rank biserial correlations ranging from 0.43 to 0.52, indicating medium to large effect sizes. These findings further justify the inclusion of these variables in the final model and may partly explain the observed model fit statistics.

## 4. Discussion

A 2018 consensus established that bruxism presents a spectrum of masticatory muscle activities, which may be classified as harmless, represent a risk factor for negative health outcomes, or even act as a potentially protective factor for positive health outcomes [5]. The interaction between masticatory muscle behaviors and other co-risk factors highlights the limitations of a simple dichotomous classification system, suggesting the need for a more nuanced, multifactorial approach to understanding bruxism and its clinical implications [5, 31, 32].

The present study evaluated awake masticatory muscle activity in adults who did not seek medical treatment due to TMD complaints before the study. Two assessment strategies were used: The oral awake behaviors from the Oral Behavior Checklist (OBC), and a real-time smartphone-based EMA assessment. The finding that 29% of the participants presented TMD-related pain symptoms aligns with previous research [33, 34]. The higher percentage of females in the TMDPS group and their increased likelihood of reporting painful TMD-related pain symptoms are also well-documented in the literature [35].

Participants in the TMDPS group demonstrated significantly more awake oral behaviors than their noTMDPS counterparts, across both assessment modes, *i.e.*, excluding BA-relaxed behavior, which showed an opposite trend. Two behaviors, *i.e.*, teeth grinding and pressing or pushing the tongue against teeth, were consistently more frequently reported in the TMDPS group in both assessment strategies. Consistent with previous findings, the broader OBC-based behavior assessment also revealed additional behaviors that were more prominent among TMDPS participants, supporting their association with TMD-related pain symptoms [4, 36]. It is noteworthy that while statistically significant, the effect size of the comparisons ranged between small to medium effects, probably due to the small sample size of the TMDPS group.

It is important to note that the study did not assess the temporal sequence between awake oral behaviors and TMD-related pain symptoms. Van Selms *et al*. [37] demonstrated that among patients diagnosed with myalgia, awake oral behaviors are positively associated with orofacial pain, but only under the condition of a strong belief of the patients in causal attribution of these behaviors to the jaw pain complaint. Although the present population included healthy adults, the possibility that participants experiencing TMD-related pain symptoms were more aware of their awake oral behaviors cannot be ruled out.

A notable finding emerged regarding the BA-relaxed behavior, which is unique to the EMA assessment. While participants with TMD-related pain symptoms initially demonstrated lower frequencies of relaxed behavior on day 1, there was a significant increase in their relaxed muscle behavior over time, with a medium-sized effect. In contrast, participants without TMD-related pain symptoms began with a relatively high frequency of BA-relaxed behavior and maintained stable levels throughout the seven-day assessment period. This finding suggests that EMA may function as a potential biofeedback tool, working as an ecological momentary intervention (EMI) rather than merely an assessment mode [21]. A recent study revealed that healthy participants maintain consistent EMA-recorded frequency levels throughout a six-month monitoring period [38]. This stability suggests that EMA may have lasting effects that extend well beyond the initial application, an interesting possibility that warrants further investigation.

When evaluating which awake oral behaviors were associated with the presence of TMD-related pain symptoms, teeth grinding came out as a significant predictive factor in the EMA assessment mode. The OBC self-report assessment mode demonstrated a higher predictive ability, with the final model showing Nagelkerke *R*^2^ of 0.468, a notably strong outcome for behavioral research involving human subjects. While this value suggests a relatively strong association between predictors and outcome, pseudo-*R*^2^ values in logistic regression models should be interpreted cautiously, particularly in small samples.

The OBC-based predictive model identified the combination of grinding, clenching, and holding or jutting the jaw as behavioral predictors of TMD-related pain symptoms. These behaviors are particularly problematic due to their prolonged duration, non-physiological movement patterns, lack of normal rest periods, sustained muscle activation, and irregular force application [32, 39].

Several key differences exist between the two assessment modes used in this study. First, the EMA assessment mode includes relaxed muscle behavior, which is absent from the OBC self-report. This allows EMA to capture not only problematic behaviors but also restorative muscle states, providing a more comprehensive behavioral profile. Second, EMA data collection is structured around mutually exclusive responses. Namely, the subject can select only one condition at a time, even though some behaviors may coexist (*e.g.*, teeth contact or teeth clenching plus pressing the tongue against the teeth). In contrast, the OBC allows for multiple behaviors to be reported simultaneously, which may result in more comprehensive reporting. However, this distinction may also lead to the underestimation of co-occurring behaviors in EMA recordings. Additionally, OBC responses may be influenced by participants’ psychological state or beliefs. For example, Van Selms *et al*. [15] showed that the likelihood of reporting higher frequencies of oral behaviors such as clenching, grinding or holding the jaw rigidly correlates with levels of depression, anxiety, and stress. Because the OBC reflects self-reported behavior over the past 30 days, it may be more susceptible to cognitive biases and emotional influences compared to the EMA’s real-time reporting, which is less prone to retrospective distortion and may more accurately reflect momentary behavior patterns.

Even though teeth grinding is among the least frequently reported awake oral behaviors [21], it emerged as a potential predictor of TMD-related pain symptoms in both models. This finding highlights the need for further investigation in clinical populations to better understand its potential role in the development or maintenance of TMD-related pain.

A dedicated EMA application, referring to five oral behaviors (relaxed, bracing, teeth contact, teeth clenching and grinding), has previously been studied in different cultural settings (*e.g.*, Portugal, Italy, Israel) [21, 40]. The present study is the first to expand the EMA protocol to include ten awake oral behaviors, adding pushing the tongue forcibly against the teeth, placing the tongue between the teeth, biting/chewing/playing with the tongue, cheeks or lips, holding or biting objects between the teeth, and chewing gum. This expanded assessment provides deeper, real-time insight into a broader range of awake oral behaviors that may be associated with TMD-related pain symptoms, particularly in a sub-clinical population.

The recently introduced Standardized Tool for Bruxism Assessment (STAB) recommends a multimodal approach for evaluating awake bruxism, combining clinical examination, single-point self-report observation, and EMA [20]. This integrated assessment strategy offers several important advantages. The OBC allows for the screening of a broader range of awake oral behaviors that (combined with certain psycho-social factors) may contribute to masticatory muscle pain and provide important data about their frequency. The broader screening also helps to raise patient awareness of awake oral behaviors, which is important for the management of possible harmful consequences. EMA’s strength lies in its ability to track the frequency of muscle relaxation over time, which was the most frequent behavior in both groups. Since achieving a more relaxed muscular condition is the primary treatment goal for TMD-related pain symptoms, this information is essential. Through the dual assessment approach, clinicians can obtain a more comprehensive understanding of their patients’ condition, enhance patient awareness, and promote relaxed behavioral patterns. As mentioned above, EMA can also serve as an EMI management tool in cases of TMD-related pain symptoms.

The Prospective Pain Evaluation and Risk Assessment (OPPERA) study estimated that approximately 4% of TMD-free adults develop a clinically confirmed first-onset of painful TMD each year [41]. This highlights the importance of early monitoring of potentially harmful awake oral behaviors, which may enable the timely implementation of preventive and therapeutic interventions. Such early intervention can help mitigate the progression of symptoms and reduce the risk of transitioning to more advanced or chronic TMD conditions [42].

It is noteworthy that the study was conducted in Israel during an ongoing war, which took its toll on the population, inducing stress and anxiety even in those not directly involved in combat. Aside from the general disruption among the civilian population, all daily routines were severely compromised, leading to an adverse impact on care for family, friends, colleagues, and other aspects of everyday life. In addition to an observed increase in teeth clenching, subjects’ awake oral behaviors were significantly associated with their ability to cope with stress adaptively (resilience coping) and their perception of stress (unpublished data). These findings underscore the potential influence of sociopolitical and environmental stressors on oral behavior patterns, and highlight the need to consider contextual psychological factors when evaluating behaviors related to TMD and bruxism.

Understanding and identifying predisposing factors is crucial for defining preventive measures that help patients and minimize the impact of potentially harmful behaviors, such as bruxism. Moreover, TMD symptoms often follow a natural course of alleviation. Subjects who participated in the present study might never seek TMD management. Clinically, subjects who have severe symptoms that need professional management are of special interest. To better understand the transition from mild, subclinical symptoms to more severe, clinically significant TMD, especially concerning awake bruxism (AB) behaviors, a prospective longitudinal study design is recommended.

Several limitations of the present study should be acknowledged. First, the study does not provide evidence of a causal relationship between awake oral behaviors and TMD-related pain. Second, despite the efforts to decrease selection bias, the study deals with university students who, while presenting a significant diversity, are not representative of the general population. Additionally, teeth grinding during awakening is one of the least frequently observed oral behaviors [21]. Therefore, its apparent association with TMD-related pain symptoms may have limited clinical relevance. Most importantly, the study did not conduct the DC/TMD Axis I clinical examination, which limits the ability to generalize findings to clinical populations with confirmed painful TMD. As such, results should be interpreted with caution when considering their applicability in clinical diagnosis or treatment planning.

## 5. Conclusions

This study explored the relationship between awake oral behaviors and TMD-related pain symptoms using both self-reported (OBC) and real-time (EMA) assessment tools in a non-clinical adult population. Key findings showed that behaviors such as teeth grinding, clenching, and jaw bracing were not only more prominent among individuals with TMD-related pain symptoms but were also significant predictors of symptom presence. Notably, EMA revealed an increase in relaxed muscle behavior over time, suggesting its potential use as both an assessment and intervention tool. Overall, the study supports a multimodal behavioral assessment approach for early identification and potential management of TMD-related symptoms.
